# Development of a tailor‐made surgical online learning platform, ensuring surgical education in times of the COVID19 pandemic

**DOI:** 10.1186/s12893-021-01203-5

**Published:** 2021-04-17

**Authors:** Sophia M. Schmitz, Sandra Schipper, Martin Lemos, Patrick H. Alizai, Elda Kokott, Jonathan F. Brozat, Ulf P. Neumann, Tom F. Ulmer

**Affiliations:** 1 Department for General, Visceral and Transplantation Surgery, University Hospital RWTH Aachen, Pauwelsstr. 30, 52074 Aachen, Germany; 2 Department of Nanomedicine and Theranostics, Institute for Experimental Molecular Imaging, RWTH University Clinic and Helmholtz Institute for Biomedical Engineering, Aachen, Germany; 3 Department of Surgery, Maastricht University Medical Centre (MUMC), Maastricht, Netherlands; 4 Audiovisual Media Center, Medical Faculty, RWTH Aachen University, Pauwelsstr. 30, 52074 Aachen, Germany; 5 Department of Medicine III, University Hospital RWTH Aachen, Aachen, Germany

**Keywords:** COVID 19, Blended learning, Flipped classroom, Surgical education, Virtual curriculum

## Abstract

**Background:**

During the worldwide COVID-19 pandemic, the quality of surgical education experiences sudden major restrictions. Students’ presence in the operating theater and on wards is reduced to a bare minimum and face-to-face teaching is diminished. Aim of this study was therefore to evaluate alternative but feasible educational concepts, such as an online-only-platform for undergraduates.

**Objective:**

A new online platform for undergraduate surgical education was implemented. A virtual curriculum for online-only education was designed.

**Methods:**

A video-based online platform was designed. Following this, a cohort of medical students participating in a (voluntary) surgical course was randomized into a test and control group. Prior to conducting a written exam, students in the test group prepared using the video platform. Students in the control group prepared with standard surgical text books. Results of the exam were used to compare educational means.

**Results:**

Students in the test group preparing through the video-based online platform reached significantly higher scores in the written exams (p = 0.0001) than students of the control group. A trend towards reduced preparation time that did not reach statistical significance was detectable in the test group (p = 0.090). Scores of “perceived workload” and “desire to become a surgeon” offered no differences between the groups. (p = 0.474 and 1.000).

**Conclusions:**

An online-only, virtual curriculum proved feasible for surgical education in undergraduates. While blended learning concepts were applied in both groups, only the test group had access to case-based videos of surgical procedures and scored significantly better in the written exams. Thus, video-based virtual education offers a realistic alternative to face-to-face teaching or conventional text books in times of restricted access to the operating theatre.

## Introduction

Surgical education offers a long history of learning through apprenticeship. Historically, surgical novices followed a skilled teacher closely to gain both knowledge of theoretical backgrounds and manual skills alike. In the last decades, novel educational concepts have emerged, but the operating theater remains the undisputed backbone of surgical education. During the ongoing COVID-19 pandemic, non-emergency operations had to be re-scheduled. Both case-numbers and in-house personal was reduced in an effort to curb infection rates [1, 2]. Furthermore, only a limited number of personnel was allowed in the operating theater, depriving students of the possibility to witness operations [1, 3–5]. These circumstances have affected surgical education on many levels- ranging from undergraduate students to senior residents [3, 6]. To continue safe surgical education, classical approaches had to shift unanticipatedly from workplace teaching to remote studying. Such new teaching concepts include e.g. the usage of podcasts, webinars, video-based reviews and online discussion in surgical communities [7–9]. As doubts on the usefulness and non-inferiority of new educational designs remain, this study aimed to evaluate a newly designed online-only curriculum for surgical education.

The concept of blended learning (employing online instruction platforms in combination with face-to-face teaching) possesses a successful history in non-surgical training [10]. One concept of it is the flipped classroom, where theoretical background is taught by online sources and the face-to-face class time is used for interactive teaching methods such as case discussion and problem solving [11–14]. Tailored to surgical specialties, this model of flipped classrooms uses video learning as a preparation for actual operations (“flipped OR”) [15]. Even video learning lacking such “flipped ORs” is well accepted among medical students and residents, as evidenced by the frequent use of video platforms (such as Youtube) for case preparations [16]. Video-based education has been described as effective and beneficial in surgical education for students and residents [17, 18]. Additionally, the availability of technical devices such as smartphones and tablets facilitates online teaching [19].

A major advantage of online education is the possibility to adapt teaching to the students’ individual demands. Settings and locations for studying are fluent, as educational videos are easily paused, restarted and repeated. Furthermore, educational methods addressing multiple processing pathways (such as auditory and visual stimuli) appear to enhance learning capabilities [9, 20].

Admittedly, increased soft skills of time management and self-motivation are required for these methods of education [4], which is either regarded a challenge or benefit of blended learning methods. As self-regulated learning strategies are gaining importance in the field of medical education [21], such developments are desirable. In all concepts of blended learning, students’ engagement appears to be crucial [3, 10, 13].

While a global health crisis such as the ongoing COVID-19 pandemic imposes major challenges to the clinical and academic field of medicine, it might also provide opportunities for educators to develop and employ new concepts of teaching and training [1, 4]. For surgery the transformation of apprenticeship-style learning towards video-based online education appears to be especially difficult, as the conventional OR experience is non-replaceable. Despite this, blended learning concepts offer a valid chance to address the lack of feedback and supervision that has been reported in the disruption of clinical clerkships during COVID-19 pandemic [4].

Hence, collaborating with the audiovisual media center of the medical faculty we developed an interactive online platform. The curriculum was designed through employing the concept of blended learning, with interweaved text-based education and virtual, supervised tutorials. The platform was based on representative surgical cases and procedures.

Hypothesizing superiority of the new platform, exam performance of different study groups was analyzed to evaluate our platform.

## Methods

### Study design

Prior to the start of the experiment, students were randomly assigned to two groups. We stratified students by their year of study and assigned them into either video learning (experimental group) or text-based education (control group). Data were pseudonymized by means of student number and stratified according to the year of study. Consequentially, students were assigned a random number by the random number function in excel. This number was consequentially used to assign the group. There were 58 students enrolled initially, 29 were assigned to the control group and 29 to the experimental group.

**Fig. 1:**
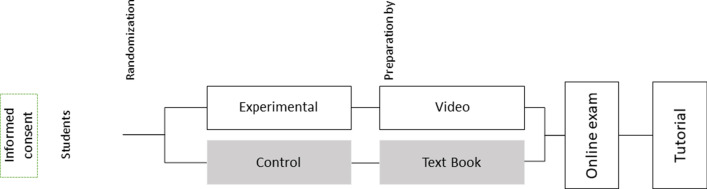
Design of a virtual curriculum for undergraduate surgical education. Course preparation included video-based preparation in the experimental group and text book-based preparation in the control group. A written exam was taken by both groups followed by an online tutorial the next day. Time for preparation was one week for both groups

In total, seven educational sessions were performed. Each session was related to a relevant surgical topic (gallbladder, thyroid, liver, inguinal hernia, stomach, and colorectal surgery). Due to students demands, a single extra session was organized discussing basic surgical principles and concepts. Seven days prior to the online session, the control group received a book chapter relating to the topic and the experimental group was granted access to our online platform. The curriculum is described in detail below and visualized in Fig. 1. To ensure sustainable clinical education all students were granted access to all videos and materials at the end of the study.

Informed written consent was obtained from all students participating in the session as well as from patients whose surgeries were recorded.All methods were carried out in accordance with relevant guidelines and regulations.The study protocol was approved by the medical ethics committee of the University Hospital Aachen (Registration Number: EK431/19).

### Online platform and virtual curriculum

An interactive online platform was developed in order to teach operative techniques and skills. For this purpose, surgical procedures were videorecorded in our operating theater and processed in order to design an interactive video format. Additionally, all students received information about the clinical case and relevant medical background information by means of standard textbooks. The excerpts from the textbooks were of 5–8 written pages.

Students received access to the text books or the video platform a week prior to the online tutorial for preparation (See Fig. 1). One day before the tutorial an online exam consisting of ten multiple choice questions was performed assessing the level of comprehension. During the online exam, students were asked to provide information on their preparation time and experienced workload. Length of the online-tutorial sessions was 60 min. During the online-tutorial sessions three visceral surgeons discussed the exam in depth and the video was watched with both the control and experimental group. There was no face-to-face teaching in any of the groups.

Before the start and at the end of the virtual curriculum a general assessment took place investigating the students’ interest in surgery and the likelihood of becoming a surgeon. Ratings took place on a continuous scale ranging from 1 to 100. Furthermore, the time students spent for preparation was assessed in a self-reporting manner.

### Statistical analysis

We calculated the following parameters: the percentage of correct, incorrect and ‘don’t know’ choices, the time spent preparing the session, the average rating displaying the likelihood of becoming a surgeon, and the adequateness of the overall workload.

For each student, the percentage of correct answers was calculated for each session. Data are represented as mean and standard error of the mean [mean ± SEM].

Outliers were excluded if values reached a value of more or less than two standard deviations from the mean.

Variables were tested for normality using Kolmogorov–Smirnov test. Quantitative data lacking normal distribution was analyzed by means of Mann-Whitney U testing. All other data was analyzed by an independent t-test. P values below 0.05 were considered statistically significant. Data was analyzed using SPSS Statistics (Version 26, IBM, Armonk, NY). Figures were created in GraphPad Prism Version 8.

## Results

Of the 58 students initially randomized, 44 (forty-four) students (75% female) completed the study. In the control group, data from 23, in the test group, data from 21 students remained for analysis. Most students (82%) were in their second year of study.

**Fig. 2:**
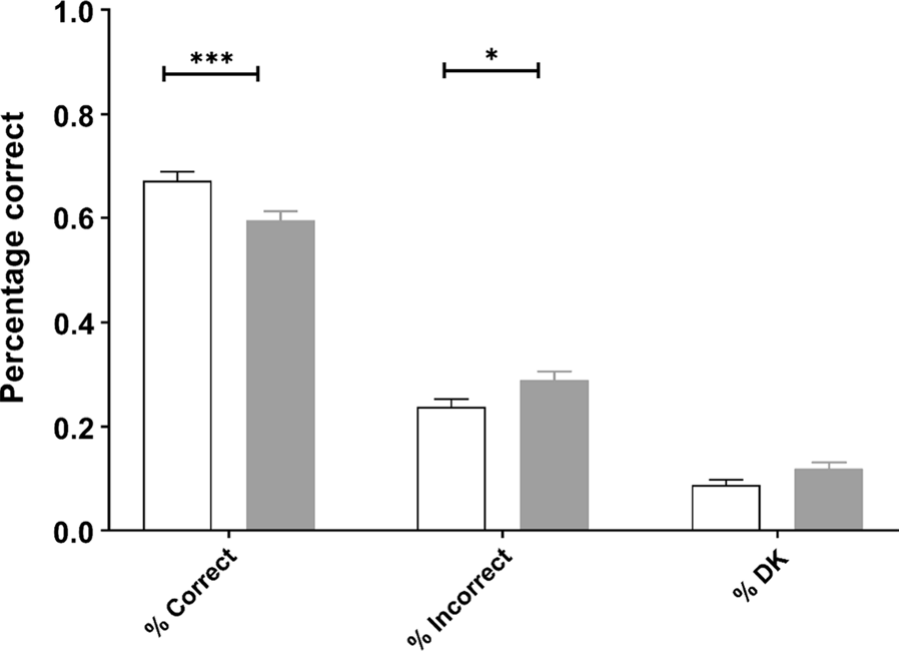
Percentage correct, incorrect and ‘don’t know’ (DK) choices for the experimental (white) and control group (grey). There was a significantly higher percentage correct in the experimental group compared to control group as well as a significantly lower percentage incorrect choices. *: p = – 04, ***: p = 0.0001

Overall, values for “correct”, “incorrect” and “don’t know” exam choices (percentages) were unequally distributed, according to Kolmogorov-Smirnov testing (p < 0.0001). Students in the video group had a significantly higher percentage of correct choices (Experimental group: 0.67 ± 0.02 vs. control: 0.60 ± 0.02; p = 0.0001, see Fig. 2) and a significantly lower number of incorrect choices (Experimental group: 0.24 ± 0.19 vs. control: 0.29 ± 0.223; p = 0.04, see Fig. 2). A statistical trend was shown for less ‘don’t know’ choices in the video group (p = 0.065), even though statistical significance was not reached.

**Fig. 3:**
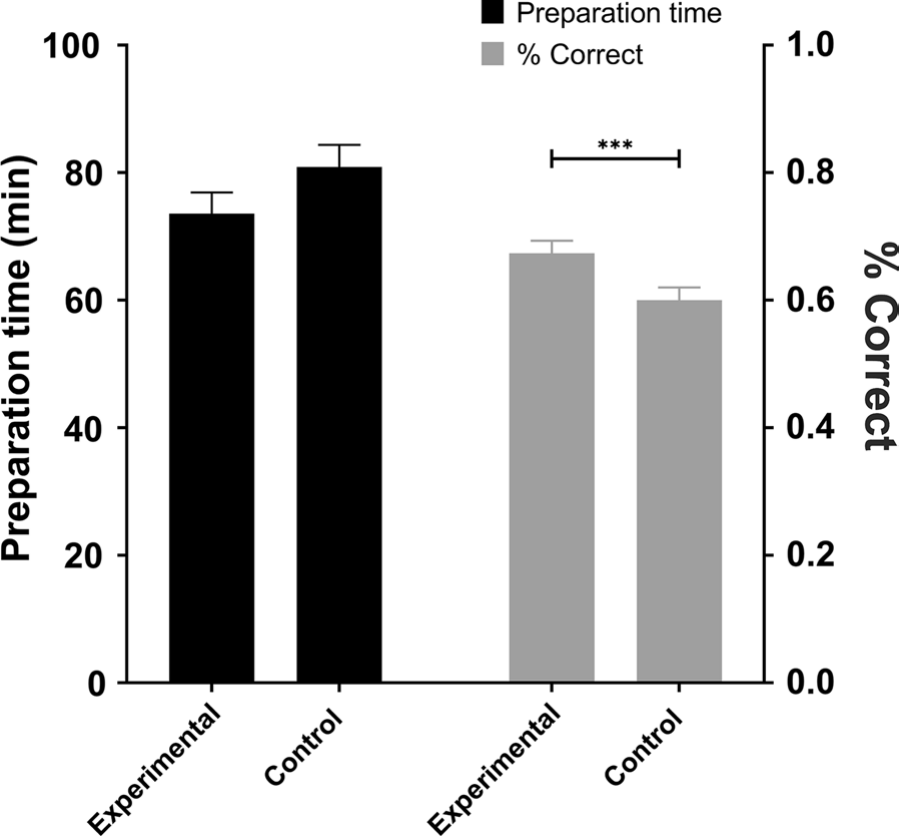
In this figure the mean preparation time in minutes (left axis, black bars) and the according percentage of correct choices (right axis, grey bars) is shown for the experimental and the control group. There was a significant difference in the percentage of correct choices between the experimental and control group (Significance bar not shown). ***: p = 0.0001

While also scoring better, a trend towards less “time spent preparing” was noticeable for students in the video group, while statistical significance was not reached (Experimental group: 74 ± 3 vs. control: 81 ± 3 min; p = 0.09, see Fig. 3).

There was a significant correlation between the time spent for preparation and the percentage of correctly provided answers in the control group (r(165) = 0.215, p = 0.006) but not the experimental group (r(158) = 0.149, p = 0.062).

**Fig. 4:**
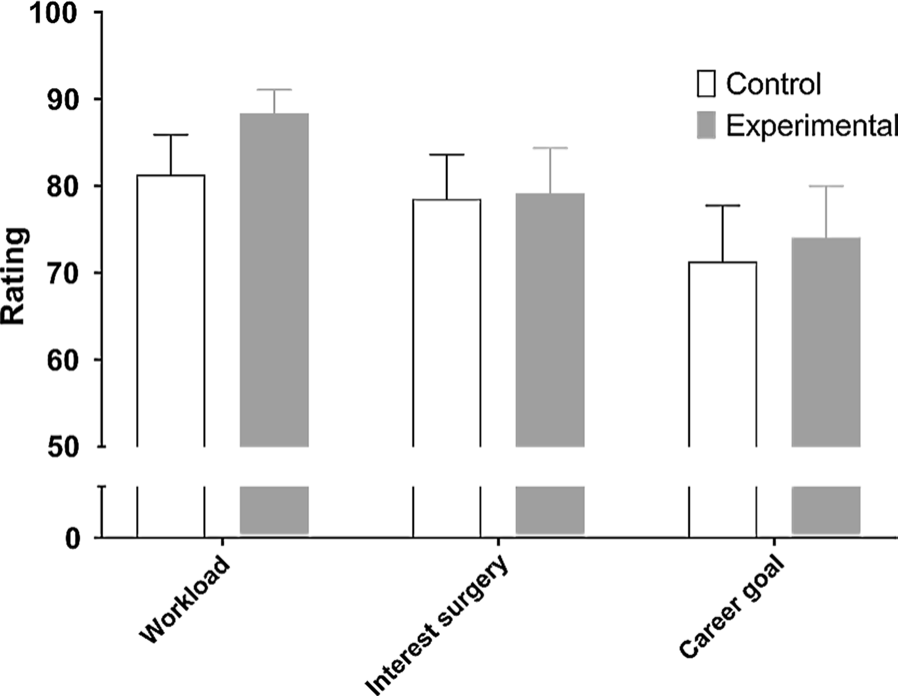
In this figure the post-interventional workload, rating for the interest in surgery, and the career goal are displayed for the two groups. Rating was performed with a score ranging from 0 (lowest) to 100 (highest). There were no statistically significant differences between control and experimental group for any of these items

We also assessed whether students experienced the time and effort they had to invest as being adequate. Additionally, we assessed the likelihood that students express the desire to become a surgeon after the online curricula in both groups. There was no difference in the experienced workload between the two groups (p = 0.474, see Fig. 4). Furthermore, the desire to become a surgeon was equally distributed between the two groups (p = 1.000, see Fig. 4).

## Discussion

In the ongoing COVID-19 pandemic, existing surgical education curricula appear inept, as they are mainly based on directly observing operations or practical experiences. Therefore, alternatives for surgical education had to be developed rapidly [1, 2, 7, 8]. The aim of this study was to evaluate the didactic value and applicability of a newly designed online-only curriculum. In order to teach better, we tailored an online-only video platform to the educational needs of undergraduate students to deliver theoretical surgical basics and operative techniques.

In the development of the online-only curriculum, concepts of blended learning and flipped classroom were applied [10–13]. While both the experimental and the control group received background information before the online tutorial, only the experimental group used interactive videos for tutorial preparation. Thus, both groups prepared with a fixed curriculum. The interactive videos provided were the only, and therefore quantifiable intervention.

Our results demonstrate that students using innovative blended learning techniques in digital format are able to compete with regular text book educated colleagues. Indeed, students using our digital means for education were able to score significantly higher in the weekly questionnaires. The experienced work load remained comparable in both groups while students in the experimental group provided better final outcomes. We are thus able to show, that in this study, online video preparation was superior to text book studying.

With the help of a tailored online platform we were further able to tackle serious challenges in medical education, not only during the COVID19 pandemic: (1) The interactive design of the videos as well as the written tests in the elective curriculum provided a possibility for assessment of students’ knowledge [3, 4]. (2) The concept of a written exam followed by a live online tutorial encouraged students’ cooperation and engagement. Mixing different means of content delivery and engaging students through tasks has been described as a key factor for successful blended learning. [10, 17, 22]. We further prove their application in this current study. (3) The application of long-distance learning ensured educational continuity while simultaneously reducing the risk of community transmission [19].

Video case preparation has been reported to be effective for undergraduates, while there are divergent results for experienced surgeons [17, 23]. However, strategies and curricula involving video-based education vary widely [17]. In our study, we included video-based preparation time followed by a written exam and an online tutorial. Our online-only curriculum incorporates features of blended learning and “flipped classrooms”, conducting theoretical learning prior to the actual class or tutorial. This way, face-to-face tutorials can focus directly on practical skills and problem solving. Similar concepts have been successfully proposed in fields such as otolaryngology, oral, and maxillofacial surgery [4, 15]. Other authors show, that a video-based, flipped classroom curriculum for residents can lead to significantly improving skill sets [14]. In line with the previously published studies, the time spent in face-to-face teaching was successfully replaceable through an online substitute.

Cautionary voices raise the question, that the employment of new media might include the lack of peer review, privacy issues and poor educator skills [24]. Additionally, existing high-quality online platforms often require expensive subscriptions, which provide a challenge for low-income students [25]. Hence, inequality in the educational sector might prosper.

All of these issues could be successfully addressed in our online-only curriculum except a preceding peer review process of the videos. In contrast, to ensure quality of the educational videos, they were discussed in detail by three experienced surgeons. Privacy issues were addressed by gathering informed written consent from the patients beforehand. The educators were trained and assisted by the audiovisual media center from our faculty. Finally, usage of the online platform was offered free of charge to any student from our faculty.

However, there are some limitations worth noting. Whether an online-only curriculum for undergraduates can meet demands of high-quality surgical training remains to be explored. Long term effects of missing OR experience cannot be assessed yet. Neither can we state with absolute certainty, that students in the control group did not use other methods of preparations. Nevertheless, as healthcare workers’ and undergraduates’ safety and wellbeing are major requirements in these times, remote solutions like ours have to be applied [6].

Coherently, the COVID-19 pandemic will not be the last challenge for medical and surgical education [1], but it might stimulate the development of novel approaches in remote learning for the future [1, 8].

To sum up, our online-only surgical curriculum could successfully be implemented and objectively ensured reasonable and rewarding undergraduate surgical education for future generations of students.

## Conclusions

Sustainable surgical education during the ongoing COVID-19 pandemic required transformation of surgical education into a new, mainly remote design. Here, we report on the development of an online-only curriculum education that uses blended learning as well as a flipped-classroom concept. A video platform was tailor made to enhance class preparation. Students scored significantly higher in weekly exams when using the newly designed online video platform than when preparing with regular text books. The workload experienced was comparable. An online-only surgical curriculum proved as an apt short-term replacement for surgical ward and OR rotations. It thus might prepare the grounds for establishing new media and blended learning concepts in future surgical education.

## Data Availability

The datasets used and/or analysed during the current study are available from the corresponding author on reasonable request.
